# Decreased Hsp90 expression in infiltrative lobular carcinoma: an immunohistochemical study

**DOI:** 10.1186/1471-2407-10-409

**Published:** 2010-08-06

**Authors:** Flora Zagouri, Theodoros Sergentanis, Afrodite Nonni, Christos Papadimitriou, Anastasia Pazaiti, Nikolaos V Michalopoulos, Panagiotis Safioleas, Andreas Lazaris, George Theodoropoulos, Effstratios Patsouris, George Zografos

**Affiliations:** 1 Breast Unit, 1stDepartment of Propaedeutic Surgery, Hippokratio Hospital, School of Medicine, University of Athens, Athens, Greece; 2 Department of Clinical and Therapeutics, Alexandra Hospital, School of Medicine, University of Athens, Athens, Greece; 3 Department of Pathology, School of Medicine, University of Athens, Athens, Greece

## Abstract

**Background:**

Elevated Hsp90 expression has been documented in breast ductal carcinomas, whereas decreased Hsp90 expression has been reported in precursor lobular lesions. This study aims to assess Hsp90 expression in infiltrative lobular carcinomas of the breast.

**Methods:**

Tissue specimens were taken from 32 patients with infiltrative lobular carcinoma. Immunohistochemical assessment of Hsp90 was performed both in the lesion and the adjacent normal breast ducts and lobules; the latter serving as control. Concerning Hsp90 assessment: i) the percentage of positive cells and ii) the intensity were separately analyzed. Subsequently, the Allred score was adopted and calculated. The intensity was treated as an ordinal variable-score (0: negative, low: 1, moderate: 2, high: 3). Statistical analysis followed.

**Results:**

All infiltrative lobular carcinoma foci mainly presented with a positive cytoplasmic immunoreaction for Hsp90. Compared to the adjacent normal ducts and lobules, infiltrative lobular carcinoma exhibited a statistically significant decrease in Hsp90 expression, both in terms of Hsp90 positive cells (%) and Allred score (74.2 ± 11.2 *vs*. 59.1 ± 14.2 p = 0.0001; 7.00 ± 0.95 *vs*. 6.22 ± 1.01, p = 0.007, Wilcoxon matched-pairs signed-ranks test). Concerning the intensity of Hsp90 immunostaining only a marginal decrease was noted (2.16 ± 0.68 *vs*. 1.84 ± 0.63, p = 0.087, Wilcoxon matched-pairs signed-ranks test).

**Conclusion:**

ILC lesions seem to exhibit decreased Hsp90 expression, a finding contrary to what might have been expected, given that high Hsp90 expression is a trait of invasive ductal carcinomas.

## Background

Hsp90 is an abundant protein in mammalian cells [1]. It forms several discrete complexes, each containing distinct groups of cochaperones that assist protein folding and refolding during stress, protein transport and degradation [2]. Hsp90 interacts with a variety of proteins that play key roles in breast neoplasia; including estrogen receptors (ER), tumor suppressor p53 protein, angiogenesis transcription factor HIF-1alpha, antiapoptotic kinase Akt, Raf-1 MAP kinase and a variety of receptor tyrosine kinases, such as erbB2 (reviewed in [3]).

Elevated Hsp90 expression has been documented in breast ductal carcinomas [4-6] as contributing to the proliferative activity of breast cancer cells. Hsp90 overexpression has been proposed as a mechanism through which breast cancer cells become resistant to various stress stimuli [6]. In this context, higher Hsp90 expression may represent a marker of poor prognosis [7]. Given the above, it would appear that pharmacological inhibition of Hsps can provide therapeutic opportunities in the field of cancer treatment [8-12]; 17- allylamino, 17-demethoxygeldanamycin (17-AAG), the first Hsp90 inhibitor to undergo clinical development, has yielded promising results [3,13]. As the above demonstrates, a wide variety of studies on Hsp90 expression in breast cancer have emerged; nevertheless, there is a marked scarcity of data on Hsp90 expression in lobular neoplasia/infiltrative lobular carcinomas in particular.

Contrary to what might have been expected, our previous work in lobular neoplasia (LN) has demonstrated downregulation of Hsp90, in respect to both the level of Hsp90 intensity and Allred score [14]. According to the most recent WHO classification, LN includes the designations atypical lobular hyperplasia (ALH) and lobular carcinoma in situ (LCIS) and refers to the entire spectrum of atypical epithelial proliferation originating in the terminal ductlobular unit, with or without involvement of ducts [15]. Nowadays, it is widely known that LN represents a risk factor and a non-obligatory precursor for the subsequent development of invasive carcinoma in either breast, of either ductal or lobular type [16].

This study aims to go beyond LN, assessing Hsp90 expression in infiltrative lobular carcinomas (ILC). The examination of ILC lesions may subsequently prove to have significant implications for the viability of Hsp90 inhibitors in breast lobular lesions.

## Methods

This study involved formalin-fixed, paraffin-embedded tissue specimens from 32 patients with ILC. The patients' age at operation ranged between 35 and 74 (median age: 53 years); information regarding the patients' clinicopathological features was also retrieved. The diagnosis of ILC was established by vacuum-assisted breast biopsy, excisional breast biopsy, lumpectomy and modified radical mastectomy. Cases of ILC coexisting with atypical ductal hyperplasia, ductal carcinoma in situ, or invasive ductal carcinoma were excluded.

Hsp90 was immunohistochemically detected using the mouse monoclonal antibody Hsp90 (clone JPB24, NCL-Hsp90, Novocastra supplied by Menarini). The dilution was 1:500 and the incubation time was 18 h (at 4°C). The visualization was performed using the Dako Envision kit. Antigen retrieval was achieved in 0.01 M citrate buffer (pH = 6.0) at 85°C for 15 min. Immunohistochemical assessment of Hsp90 was performed both in the lesion and the adjacent normal breast ducts and lobules, the latter serving as control. Negative controls were assessed by omitting the primary antibody.

Concerning Hsp90 assessment: i) the percentage of positive cells and ii) the intensity were separately analyzed. Subsequently, the Allred score was appropriately calculated (Table 1) [17,18].

**Table 1:** Algorithm for the calculation of the Allred score [17,18].

Observed values	"Allocated" values for the calculation of the 0-8 Allred score
Percentage of positive cells	
*None*	0
*<1%*	1
*1% to 10%*	2
*10% to 33.3%*	3
*33.3% to66.7%*	4
*more than 66.7%*	5

Intensity of staining	
*weak*	1
*intermediate*	2
*strong*	3

For immunohistochemistry (IHC), the following antibodies were used: PgR (636, Dako), ER (ID5, Dako) and c-erbB-2 (CB11, Novocastra™). Sections (4 μm thick) cut from formalin-fixed paraffin embedded tissue were used. After deparaffinization in xylene and hydration in graded ethanol solutions, the sections of breast carcinoma tissue were subjected to pretreatment in order to enhance antigen retrieval. The EnVision + System-HRP (DAB) (DakoCytomation, Glostrup, Danemark) was used with primary antibodies against the following antigens: PgR, ER and c-erbB-2. Immunohistochemistry was performed according to the protocols provided by the manufacturer. Concerning the immunohistochemical expression of ER and PR, both the intensity (negative, 1+ to 3+) and percentage of immunopositive cells were evaluated. Subsequently, the Allred score was calculated [16].

The expression of c-erbB-2 was assessed as follows: i) negative, when no staining was documented or when membrane staining was present in less than 10% of tumor cells, ii) weak staining (+), when partial membrane staining was documented in more than 10% of tumor cells, iii) moderate staining (++) when weak/moderate complete membrane staining was present in more than 10% of tumor cells and iv) strong staining when strong, complete membrane staining was observed in more than 10% of tumor cells. Cases with negative and weak c-erbB2 staining were considered as negative, whereas cases with strong c-erbB2 staining were considered as positive. In cases with moderate staining, CISH was performed; subsequently these cases were considered as negative or positive.

In all cases, ten fields (×40 magnification) were assessed and a minimum of 100 cells were evaluated in the designated areas, so as to appraise the lesion as a whole. The immunohistochemical evaluation was performed independently by two consultant histopathologists (AN and AL).

Kappa statistic was performed to assess inter-rater agreement at both the estimation of Hsp90 percentage and intensity; for the optimal interpretation of the result, it should be kept in mind that pathologists rated Hsp90 percentage at an increment of 5% i.e., possible values are: 60%, 65%, 70% and so forth.

The intensity was treated as an ordinal variable-score (0: negative, low: 1, moderate: 2, high: 3). Regarding Allred score and Ki-67 percentage, all numbers are provided as mean ± SD. Two main analyses were performed: i. comparison of Hsp90 Allred score between the lesion and the adjacent normal ducts and lobules and ii. evaluation of the associations between Hsp90 Allred score and ER positivity, progesterone receptor (PR) positivity, c-erbB2 status and Ki-67 (%). Due to deviation from the normal distribution, non-parametric statistics were chosen. The statistic performed in each case is mentioned in parentheses in the text. Where appropriate, power calculations are presented. Statistical analysis was performed with STATA 8.0 statistical software (Stata Corporation, College Station, TX, USA).

Informed consent was obtained from all participants in this study. This study has been approved by the local Ethics Committee, in accordance with the Helsinki Declaration.

## Results

**Figure 1:**
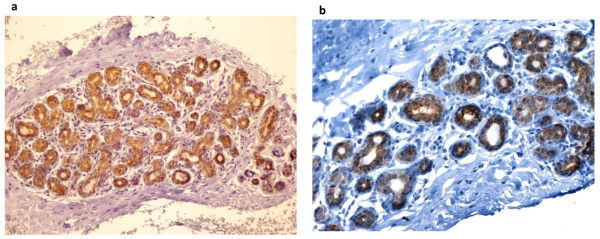
**Normal TDLU with strong Hsp90 immunostaining (×200) (Figure 1a, 1b)**.

**Figure 2:**
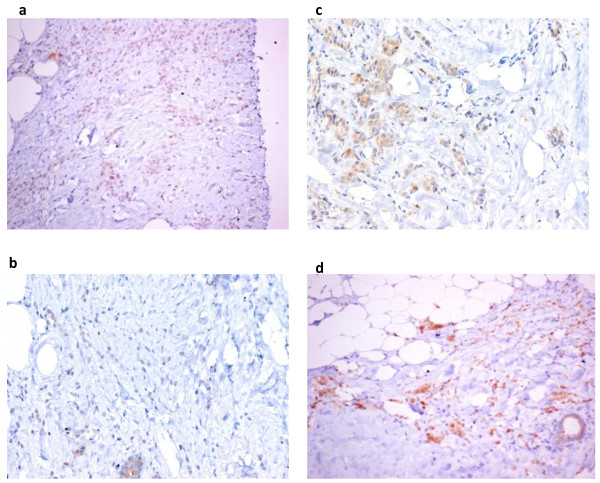
**Infiltrative lobular carcinoma with slight Hsp90 immunoreactivity (×200) (Figure 2a, 2b, 2c) and with moderate Hsp90 expression (×200) (Figure 2d)**.

The description of the study sample is presented in Table 2. Hsp90 exhibited mainly cytoplasmic immunoreactivity in epithelial cells of normal breast (ducts and lobules) (Figure 1), and ILC (Figure 2). Some epithelial cells of ILC also showed nuclear staining; nevertheless, all ILC foci mainly presented with a positive cytoplasmic immunoreaction for Hsp90. The positive cell percentage and the staining intensity were evaluated.

**Table 2:** Characteristics of patients and histological features of invasive lobular carcinomas.

Categorical Variables	Frequency (%)
Menopausal status	
*Premenopausal*	8 (25.0)
*Postmenopausal*	24 (75.0)
First-degree relative with breast cancer	
*Yes*	6 (18.8)
*No*	26 (81.2)
Breastfeeding	
*Yes*	24 (75.0)
*No*	8 (25.0)
Grade	
*1*	5 (15.6)
*2-3*	27 (84.4)
Tumor diameter	
<2 cm	6 (18.8)
≥2 cm	26 (81.2)
Nodal status	
*Positive*	8 (25.0)
*Negative*	24 (75.0)
Metastasis	
*Yes*	0 (0.0)
*No*	32 (100.0)
ER status	
*Positive*	27 (84.4)
*Negative*	5 (15.6)
PR status	
*Positive*	25 (78.1)
*Negative*	7 (21.9)
c-erbB2 status	
*Positive*	7 (21.9)
*Negative*	25 (78.1)

**Continuous variable**	**mean ± SD**

Ki-67 (%)	12.0 ± 5.1

The percentage of Hsp90 positive cells, the intensity of Hsp90 staining, as well as the Allred score are presented in detail in Table 3 and Table 4. Concerning ILC, the inter-observer agreement was 90.6% for Hsp90 intensity (kappa = 0.8289, p < 0.0001, rejecting the null hypothesis of random ratings) and 81.3% for Hsp90 percentage (kappa = 0.7780, p < 0.0001, rejecting the null hypothesis of random ratings).

**Table 3:** Expression of Hsp90 in invasive lobular carcinomas (ILC) and the normal adjacent ducts and lobules (mean ± SD).

	Adjacent normal ducts and lobules	ILC	**p**^**a**^
**Hsp90 intensity (score)**	2.16 ± 0.68	1.84 ± 0.63	**0.087**
**Hsp90 positive cells (%)**	74.2 ± 11.2	59.1 ± 14.2	**0.0001**
**Hsp90 Allred score**	7.00 ± 0.95	6.22 ± 1.01	**0.007**

^a^p-value derived from Wilcoxon matched-pairs signed-ranks test

**Table 4:** Detailed results of Allred score in invasive lobular carcinomas (ILC) and the normal adjacent ducts and lobules.

Allred score	Adjacent normal ducts and lobules	ILC
**4**	0 (0.0)	1 (3.1)
**5**	4 (12.5)	6 (18.8)
**6**	2 (6.2)	14 (43.7)
**7**	16 (50.0)	7 (21.9)
**8**	10 (31.3)	4 (12.5)

Figures are frequency (percentage).

A statistically significant decrease in Hsp90 expression, both in terms of positive cells(%) and Allred score, was noted (74.2 ± 11.2 *vs*. 59.1 ± 14.2 p = 0.0001; 7.00 ± 0.95 *vs*. 6.22 ± 1.01, p = 0.007, Wilcoxon matched-pairs signed-ranks test). The decrease in Hsp90 intensity (2.16 ± 0.68 *vs*. 1.84 ± 0.63) was of borderline statistical significance (p = 0.087, Wilcoxon matched-pairs signed-ranks test).

Null association was noted between Hsp90 Allred score and ER positivity (6.40 ± 0.55 for ER negative cases vs. 6.19 ± 1.08 for ER positive cases, p = 0.547, Mann-Whitney-Wilcoxon test for independent samples), PR positivity (6.43 ± 0.98 for PR negative cases vs. 6.16 ± 1.03 for PR positive cases, p = 0.532, same test), c-erbB2 status (6.20 ± 1.04 for c-erbB2 negative cases vs. 6.29 ± 0.95 for c-erbB2 positive cases, p = 0.904, same test) as well as Ki-67(%) (Spearman's rho = -0.083, p = 0.651).

## Discussion

To our knowledge, this is the first study to demonstrate a significantly decreased Hsp90 expression in infiltrative lobular carcinomas, both in terms of percentage and Allred score. This finding could be viewed in a wider context; the present findings are consistent with those previously described by our team on precursor lobular lesions (LN).

The persistent downregulation of Hsp90 expression throughout the whole lobular series (at the precursor and invasive components) may be contrary to what might have been expected; it is known that Hsp90 overexpression is a feature of invasive ductal carcinomas [5-7]. It is thus tempting to speculate that the whole lobular series may display a discrete, less intense profile of Hsp90 expression, which differentiates itself from the marked upregulation in ductal carcinomas.

The underlying mechanisms of this discrepancy remain elusive. ILC express c-erbB2 at a far less extent than ductal carcinomas [19]. Given that c-erbB2 has been reported to positively associated with Hsp90 expression [7], it is tempting to speculate that downregulation of Hsp90 in ILC may be a c-erbB2 related event. However, no significant association was noted between c-erbB2 status and Hsp90 Allred score in our cohort; this null, inconclusive finding should be interpreted with caution, as only seven cases were c-erbB2 positive. In other words, the small number of c-erbB2 positive cases may have blurred the association between c-erbB2 and Hsp90 at a certain extent.

The present study comes to expand the results of our previous immunohistochemical study on LN lesions. In the case of LN we have postulated that the decreased Hsp90 expression may be partly due to the self-limiting, close-to-normal character of the lesion, as well as to the relatively milder stress response when compared to cancer [14]. The present study uncovers an Hsp90 expression pattern (relative decrease) which seems applicable to the whole continuum of lobular lesions and may be thus revealing of an inherent trend of the whole breast lobular series. Nevertheless, the fact that the finding seems to persist in the invasive lesions may suggest that the proliferation advantage in lobular lesions is an Hsp90-independent process. This hypothesis seems further supported by the fact that Ki-67 expression was not associated with Hsp90 expression in infiltrative lobular carcinomas.

The downregulation of Hsp90 for the entire lobular series is a phenomenon which may be of particular clinical significance. Anti-Hsp90 drugs are currently tested in trials with very promising results [20-23]. Nevertheless, no trials have focused on lobular lesions. Consequently, it would appear that the effects of Hsp90 targeting drugs should be evaluated separately on ductal and lobular carcinomas, since the effectiveness may be limited accordingly.

In our cases, some epithelial cells of ILC showed scarce (i.e. <5%) nuclear Hsp90 localization; it should be noted that the aforementioned nuclear staining was not taken into account at the calculation of Allred score, as such a percentage may have been caused by technical reasons. Allred score was based exclusively on cytoplasmic Hsp90 staining. Nevertheless, the significance of nuclear Hsp90 expression remains elusive, as some studies have not documented any nuclear Hsp90 expression in invasive ductal carcinomas [7], as opposed to other studies [24]. This finding has already been reported in invasive breast carcinomas and has been correlated with MHC class I expression [25].

In relation to the limitations of this study, it is worth referring to a number of points. Firstly, the small study sample should be declared; as a result, the present findings need to be reproduced in larger follow-up studies, so as to thoroughly examine the robustness as well as the clinical and prognostic implications of this immunohistochemical study. Accordingly, the fact that the decrease in Hsp90 intensity was solely of borderline statistical significance may not denote an inconsistency in results, as it could reach formal statistical significance in the context of a larger sample size (larger cohort). In any event, the Allred score, integrating both percentage and intensity, confirmed the decrease despite the relatively small sample size.

Another limitation of this study pertains to the method performed (i.e. immunohistochemistry). Notwithstanding, Hsp90 downregulation might not be contestable given its clear demonstration in a relatively less sensitive assessment (immunohistochemical procedure and visual scoring). To ensure the objectivity of the assessment, the percentage and intensity were assigned by two independent pathologists blind to one another's results. Nevertheless, under the light of the above inherent limitations of the method adopted, the results need to be confirmed by other methods apart from immunohistrochemistry (such as Western blot). Indeed, in vitro mechanistic studies seem indispensable for definitive conclusions; it should be acknowledged that such mechanistic studies were not feasible in the clinical setting, in which this study was performed. Collaborative efforts overcoming such limitations seem to be needed for further substantiation of the present findings in the future.

## Conclusion

In conclusion, it can be said that Hsp90 expression seems decreased in ILC lesions compared to the normal adjacent breast ducts and lobules, a finding contrary to what might have been expected, given that high Hsp90 expression is a trait of invasive ductal carcinomas. Further studies adopting quantitative procedures, assessing mRNA levels and the pathophysiologically associated estrogen receptor status seem mandatory.

## Competing interests

The authors declare that they have no competing interests.

## Authors' contributions

All Authors have read and approved the final manuscript. Moreover, FZ: conceived the idea, participated in the design of the study and assisted in the writing of the manuscript; TS: participated in the design of the study, performed the statistical analysis and assisted in the writing of the manuscript; AN: performed the pathological evaluation; CP: evaluated critically the manuscript; AP: performed vacuum-assisted breast biopsy and open surgery and critically revised the manuscript for important scientific content; NM: performed vacuum-assisted breast biopsy and open surgery; PS: performed vacuum-assisted breast biopsy and open surgery; AL: performed the pathological evaluation; GT: performed vacuum-assisted breast biopsy and open surgery; EP: evaluated critically the manuscript; GZ: conceived of the study, participated in its design, performed vacuum-assisted breast biopsy and open surgery and evaluated critically the manuscript

## Pre-publication history

The pre-publication history for this paper can be accessed here:

http://www.biomedcentral.com/1471-2407/10/409/prepub
